# Regulating the Regulators: The Control of Transcription Factors in Plant Defense Signaling

**DOI:** 10.3390/ijms19123737

**Published:** 2018-11-24

**Authors:** Danny W-K Ng, Jayami K. Abeysinghe, Maedeh Kamali

**Affiliations:** 1 School of Life Sciences and State Key Laboratory of Agrobiotechnology, Chinese University of Hong Kong, Shatin, Hong Kong, China; 2 Department of Biology, Hong Kong Baptist University, Kowloon Tong, Hong Kong, China; 15485382@life.hkbu.edu.hk (J.K.A.); maedeh-kamali@hkbu.edu.hk (M.K.)

**Keywords:** defense, transcription factor regulations, AP2/ERF, bHLH, bZIP, MYB, NAC, WRKY

## Abstract

Being sessile, plants rely on intricate signaling pathways to mount an efficient defense against external threats while maintaining the cost balance for growth. Transcription factors (TFs) form a repertoire of master regulators in controlling various processes of plant development and responses against external stimuli. There are about 58 families of TFs in plants and among them, six major TF families (AP2/ERF (APETALA2/ethylene responsive factor), bHLH (basic helix-loop-helix), MYB (myeloblastosis related), NAC (no apical meristem (NAM), *Arabidopsis* transcription activation factor (ATAF1/2), and cup-shaped cotyledon (CUC2)), WRKY, and bZIP (basic leucine zipper)) are found to be involved in biotic and abiotic stress responses. As master regulators of plant defense, the expression and activities of these TFs are subjected to various transcriptional and post-transcriptional controls, as well as post-translational modifications. Many excellent reviews have discussed the importance of these TFs families in mediating their downstream target signaling pathways in plant defense. In this review, we summarize the molecular regulatory mechanisms determining the expression and activities of these master regulators themselves, providing insights for studying their variation and regulation in crop wild relatives (CWR). With the advance of genome sequencing and the growing collection of re-sequencing data of CWR, now is the time to re-examine and discover CWR for the lost or alternative alleles of TFs. Such approach will facilitate molecular breeding and genetic improvement of domesticated crops, especially in stress tolerance and defense responses, with the aim to address the growing concern of climate change and its impact on agriculture crop production.

## 1. Introduction

Being limited and fixed to their immediate surroundings, plants rely on developing intricate sensing and signal transduction mechanisms in defense against various external biotic and abiotic threats. Multiple components act in concert to build up the cellular signals leading to innate immunity against pathogens and stress adaptation. Morphological structures, such as trichomes, hairs, spines and waxy cuticles, form the first direct line of mechanical defense, preventing and deterring the entry of pathogens [1]. Upon invasion by pathogens or microbes, recognition of conserved pathogen-associated molecular patterns (PAMPs) and microbial-associated molecular patterns (MAMPs) by pattern-recognition receptors (PRRs) of plants induces pattern-triggered immunity (PTI) [2,3]. In addition, some plants can recognize specific effector molecules that are produced by pathogens upon infection, triggering effector-triggered immunity (ETI) [4]. Both PTI and ETI lead to activation of various signaling transduction pathways involving the mitogen-activated protein (MAP) kinase (MAPK), reactive oxygen species (ROS), salicylic acid (SA), jasmonic acid (JA), ethylene (ET), and other phytohormones and signaling molecules. These eventually result in the production of anti-microbial compounds and secondary metabolites, modification of cellular structure through callose deposition, and programmed cell death in response to the stress [5,6]. To achieve all these, transcriptional regulators form an important node to balance the trade-off between growth and defense for optimal allocation of resources and survival of plants [7]. There are already many excellent reviews reporting their induced expression as well as their importance and roles in regulating defense phytohormones and secondary metabolites biosynthesis through a complex web of factors [8,9,10,11,12,13,14,15]. Here, we review and highlight some of the added aspects related to the control of these representative transcriptional regulators at transcript and protein levels, with examples of specific members from selected transcription factor (TF) families involved in plant defense. Finally, we discuss the potential of exploring such regulations of the regulators in major crops (e.g., wheat, rice, maize, and soybean) and their wild relatives, providing insights in improving disease resistance and stress tolerance of crops in the fight against climate change.

## 2. The Six Major Transcription Factor Families in Plant Defense

**Figure 1:**
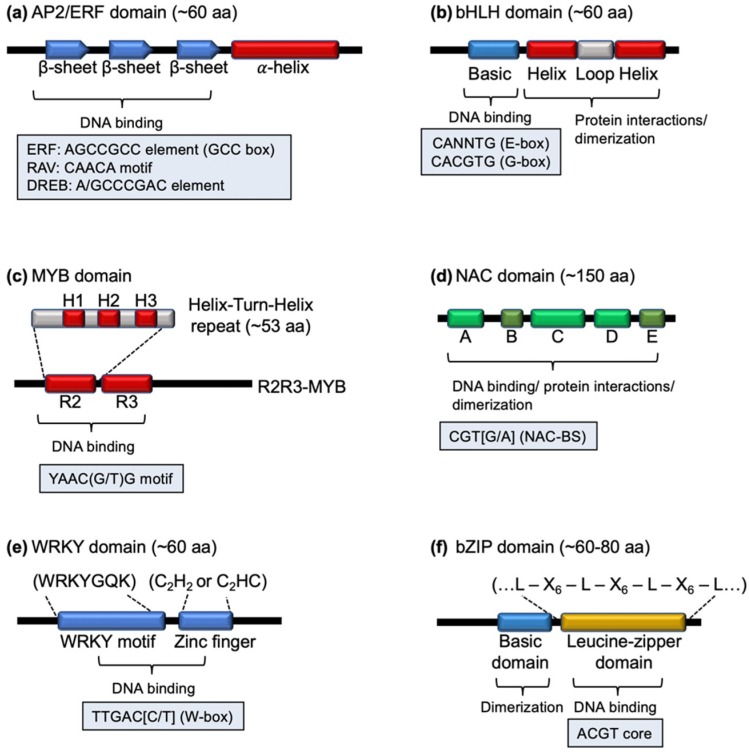
Schematic diagram of the representative domain of the six families of transcriptional regulators involved in plant defense. (**a**) AP2/ERF domain with three β-sheet strands for DNA binding and an α-helix motif. (**b**) bHLH domain with an N-terminal basic domain for DNA binding, followed by a helix-loop-helix motif that functions in protein-protein interactions or dimerization. (**c**) MYB domain formed by the presence of one-four helix-turn-helix repeat(s) involve in DNA binding. Different subclasses are characterized by having one-four copies of the repeat. The R2R3-MYB class is shown. (**d**) NAC domain consisting of 5 sub-domains, three highly conserved (A, C, D) and two diverse (B, E) sub-domains. (**e**) WRKY domain characterized by having a highly conserved WRKY motif followed by a zinc finger with either a C_2_H_2_- or C_2_HC-type zinc finger. (**f**) bZIP domain containing an N-terminal basic domain for protein dimerization, which is followed by a leucine zipper region containing up to nine heptad repeats. The sequence of typical DNA target for each family of TFs are indicated in the boxes. aa: amino acids; ERF: ethylene responsive factor; RAV: related to abscisic acid insensitive3 (ABI3)/viviparous1 (VP1); DREB: dehydration-responsive element-binding protein; H1: helix 1; H2: helix 2; H3: helix 3; R2: repeat 2; R3: repeat 3; NAC-BS: NAC binding site; L: Leucine; X_6_: any six amino acid residues.

Among the 58 identified families of transcription factors (TFs) in higher plants [16], six TF families are closely related to defense signaling with overlapping roles in mediating various signaling cascades in PTI and ETI. These six TF families are: AP2/ERF (APETALA2/ethylene responsive factor), bHLH (basic helix-loop-helix), MYB (myeloblastosis related), NAC (no apical meristem (NAM), *Arabidopsis* transcription activation factor (ATAF1/2), and cup-shaped cotyledon (CUC2)), WRKY, and bZIP (basic leucine zipper) [17,18] (Table 1; [16]). These TF families are classified based on the presence of a conserved DNA binding domain (Figure 1) as well as their unique and overlapping roles in plant development and defense.

### 2.1. The AP2/ERF Family

The AP2/ERF family of transcription factors makes up one of the largest group of TFs involved in abiotic stress responses in plants [19]. Factors within this family are characterized by the presence of an AP2/ERF DNA binding domain that consists of three β-sheet strands (responsible for DNA binding) followed by an α-helix motif [20] (Figure 1a). Based on the variation and the presence of conserved motifs outside the AP2 domain, several subfamilies with different DNA affinities were identified, including AP2, ERF, RAV (related to abscisic acid insensitive3 (ABI3)/viviparous1 (VP1)) and DREB (dehydration-responsive element-binding protein) subfamilies [19,21]. Each of these subfamily members recognizes different cis-elements and possesses additional specific conserved motifs that are required for other molecular functions, such as protein-protein interaction and phosphorylation [19].

### 2.2. The bHLH Family and MYB Families

The bHLH and MYB families represent two other large groups of TFs to which members from these two families can form complexes; parallel functional evolution has been described between these two families [22]. The bHLH TFs contain a basic-helix-loop-helix (bHLH) domain with the N-terminus basic DNA binding domain region and the C-terminus protein interaction domain region [23] (Figure 1b). Within this TF family, MYC (myelocytomatosis related) represents one of the major classes of bHLH factors in mediating jasmonate-responsive gene expression [24]. The other large group of TFs is the MYB family proteins that are characterized by having up to four repeat sequences (R), each containing three α-helices (H1, H2, and H3). Within each repeat, the H2 and H3 helices form a helix-turn-helix structure that is responsible for DNA interaction of the MYB domain (Figure 1c). Depending on the number of repeats, the MYB family proteins can be classified into four different classes, R1, R2R3, R3 and R4 [25]. Among these four classes, the R2R3 class of MYB proteins is plant specific, and forms the largest group of MYB TFs functioning in response to biotic and abiotic stresses as well as primary and secondary metabolisms [10,26].

### 2.3. The NAC Family

NAC family proteins contain a conserved N-terminus DNA binding domain and a C-terminus activation domain. The DNA binding domain of NAC factors is found originally in the NAM, ATAF1, ATAF2 and CUC2 factors. In general, the conserved NAC domain consists of five sub-domains, three highly conserved A, C, and D sub-domains and two diverse B and E sub-domains, among the NAC family of TFs [27] (Figure 1d). In addition to DNA binding, the NAC domain is involved in nuclear localization and homo- and hetero-dimer formation through interaction with other NAC domain-containing proteins [28].

### 2.4. The WRKY and bZIP Families

Both the WRKY and bZIP protein families regulate diverse developmental and stress-related processes. The WRKY family proteins are defined by the presence of a 60 amino acid-long WRKY DNA binding domain consisting of four β-sheet strands, forming the WRKY motif with a typical WRKYGQK sequence followed by a zinc finger motif (Figure 1e). Depending on the number of WRKY domains and the structure of the zinc finger motif (C_2_H_2_ or C_2_HC), three main groups of WRKY factors can be identified [13]. bZIP family proteins are characterized by having a basic region for DNA binding and a leucine zipper region for protein dimerization (Figure 1f). The leucine zipper domain contains up to nine heptad repeats with the presence of a leucine in every seven amino acids, thereby allowing two monomers to ‘zip up’ when forming the protein dimer [29]. It is believed that while auto- and cross-regulations are important in regulating WRKY expression, protein dimerization through the C-terminal leucine zipper domain of bZIP factors forms an additional regulatory node for this family of TFs [30].

## 3. The Regulation of the Regulators at the Transcript Level

**Figure 2:**
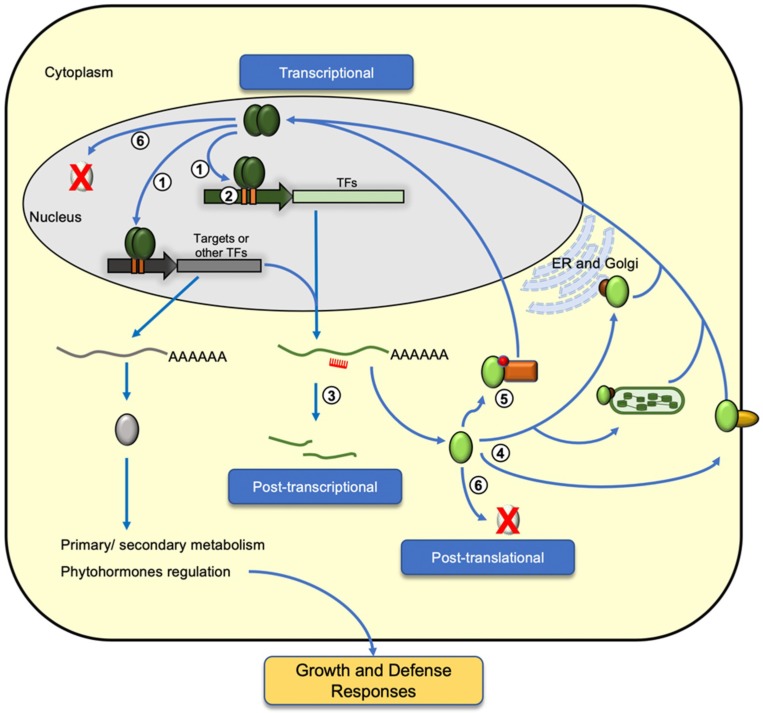
The regulation of transcriptional regulators in defense. Diagram illustrates the regulation of defense transcription factor (TF) family members at different levels. At the transcriptional level, auto- and cross-regulation among members of the same TF family or between members of different TF families forms a regulatory node for their expression (1). Epigenetic changes, such as histone modification and DNA methylation can also direct the genetic regulation and priming of the TF gene expression in response to stress (2). At the post-transcriptional level, many defense regulators are targeted by small RNAs (e.g., miRNA) for precise control of the transcript accumulation (3). In addition, the presence of a characteristic DNA binding domain (Figure 1) and various conserved domains among the TFs mediate various post-translational controls of these regulators through different mechanisms, including protein localization in intracellular membranes (4), interaction and modification with other cellular proteins in the cytoplasm (5), or ubiquitination (6). Regulation and interactions among these TF families control expression of their downstream targets involved in primary and secondary metabolisms, as well as phytohormone production, thereby mediating growth and defense in plants. ER: endoplasmic reticulum.

Expression of defense-related genes is a costly process and how transcription factors regulate their targets is not determined only by their binding to specific DNA sequences. In order to mount an efficient defense response against biotic and abiotic stresses, the level of transcription factors is also a determinant of their activity. Auto- and cross-regulation of members within the same TF family forms one of the control nodes of the defense signaling pathway. Moreover, epigenetic and post-transcriptional controls also play roles in fine-tuning their expression dynamics (Figure 2).

### 3.1. Auto- and Cross-Regulation of TFs

In response to external biotic and abiotic stresses, transcription factors function in transducing external cues into intracellular signals, thereby eliciting specific hormone signaling pathways and gene expression cascades for activation of defense-related targets. Among the six TF families involved in plant defense, activations are also subject to transcriptional regulation through auto- and cross-regulation within the same TF family (Figure 2, step 1). Many *WRKY* gene promoters containing multiple W-boxes (TTTGAC/T) are involved in auto-regulation or cross-regulation by modulating multiple stress signaling pathways [13,14,31]. WRKY TFs bind to the W-box elements in their promoter, thereby regulating their expression through auto-regulation. For example, WRKY33 is required for phytoalexin biosynthesis in response to pathogen infection in *Arabidopsis* [32,33]. In response to a pathogen challenge, AtWRKY33 is capable of binding to its promoter, thereby forming a positive feedback regulatory loop to enhance the expression of camalexin biosynthetic genes [34]. Another WRKY TF, AtWRKY18, was found to bind to the W-boxes at its promoter, negatively regulating its expression to achieve a balance between growth and defense [35]. It is common that multiple members within the same family share related and redundant roles in mediating their downstream signaling pathways, thereby cross-regulating each other at the transcriptional level. The three closely related WRKY TFs—WRKY18, WRKY40, and WRKY60—were found to cross-regulate each other in mediating abscisic acid (ABA) signaling [36]. Upon phosphate starvation, activation of WRKY45 and WRKY75 was found to upregulate the expression of the *PHOSPHATE TRANSPORTER1;1* (*PHT1;1*) while WRKY45 and WRKY75 cross-repressed each other [37,38].

Similar to the WRKY family, many NAC TFs also form extensive auto- and cross-regulatory networks in mediating both biotic and abiotic stress responses [8]. In *Arabidopsis*, ANAC013 (Arabidopsis NAC domain containing protein 13) and ANAC017 (Arabidopsis NAC domain containing protein 17) were found to be involved in the mitochondrial retrograde regulation (MRR) under oxidative stress and a cis-regulatory element (mitochondrial dysfunction motif; MDM) can be found at their promoters, suggesting a positive feedback regulation enhances their transcripts and targets expression under stresses [39,40]. In addition to their obvious roles as a color pigment, anthocyanins are implicated to play a role in plant defense against herbivores [41,42]. In *Rosaceae,* positive autoregulation of MYB10 and natural variation of a minisatellite targeting motif at the upstream regulatory regions of *MYB10* is linked to anthocyanin accumulation in different apple varieties [43,44]. In contrast, negative autoregulation forms part of the regulatory circuit for MYB4 in *Arabidopsis* and rice [45,46]. Within the AP2/ERF family, the DREB subgroup of factors is involved in abiotic stress and cross-regulation. The bHLH family factor, ICE1 (Inducer of CBF (C-repeat binding factor) Expression 1), was found to bind the cold inducible motifs at the *C-repeat binding factor 3* (CBF3; a DREB1C factor) promoter upon cold stress, activating *CBF3* expression [47]. Another bHLH factor, PIF7 (phytochrome-interacting factor 7), also cross-regulates *DREB1C* to suppress its expression through binding to the G-box at the *DREB1C* promoter [48]. In *Catharanthus roseus*, JA-induced expression of the AP2/ERF TF, ORCA3 (Octadecanoid-derivative responsive Catharanthus AP2-domain protein 3), is cross-regulated by the bHLH TF, CrMYC2 [49]. Unlike the other TF families, reports on auto- and cross- regulation of the bZIP family of TFs are limited.

### 3.2. Epigenetic and Post-Transcriptional Regulation of TFs

Expression of biotic stress-responsive genes in *Arabidopsis thaliana* is regulated by DNA methylation and demethylation [50]. In rice, exogenous application of 5-azadeoxycytidine (DNA methylation inhibitor) led to enhanced resistance against *Xanthomonas oryzae* pv*. oryzae* (*Xoo*) in the progenies of the treated plants. Such observed phenotypes were found to correlate with hypomethylation at the *Xa21G locus* and expression of the Xa21G protein in the *Xoo*-infected line, suggesting transgenerational inheritance of hypomethylation and resistant traits in the progeny [51]. Another study found that mutation of DNA demethylases in *Arabidopsis* enhances its susceptibility to the fungal pathogen, *Fusarium oxysporum*, and that DNA demethylases positively modulate the expression of stress responsive genes involving fungal infection [52]. In addition to DNA methylation, histone acetylation and methylation form another level of regulation for responses against environmental stress and pathogens (Figure 2, step 2). Many studies have also reported histone modification as a form of epigenetic memory in mediating the priming and transgenerational inheritance of defense-related genes for a rapid response against recurring stresses [53,54,55]. In systemic acquired resistance (SAR), *AtWRKY6* and *AtWRKY29* loci were primed with H3K4 (histone H3 lysine 4) dimethylation and H3K14 acetylation for increased expression upon challenge inoculation of pathogens [56]. In *Arabidopsis*, WRKY70 forms one major node in modulating the crosstalk between salicylic acid (SA) and jasmonic acid (JA) signaling pathways [57]. Expression of WRKY70 is epigenetically regulated by the *Arabidopsis* homolog of Trithorax (ATX1) through H3K4 trimethylation at the *WRKY70* locus [58]. In maize, ZmNAC111 is a positive regulator for drought tolerance, and RNA-directed DNA methylation and H3K9 dimethylation at the *ZmNAC111* promoter is responsible for its repression, hence sensitivity to drought stress in temperate maize germplasm [59].

At the post-transcriptional level, evidence is accumulating to show that microRNAs (miRNAs) play important roles in plant stress responses through targeting the defense-responsive TFs (Figure 2, step 3). Under abiotic stress, ABA (abscisic acid)-induced accumulation of miR159 was found to mediate degradation of *MYB33* and *MYB101* transcripts in *Arabidopsis*, to which they are positive regulators of ABA responses [60]. The NAC domain transcription factor is negatively regulated by miR164 [61,62]. In wheat (*Triticum aestivum* L.), *TaNAC21*/22 is a negative regulator of response against stripe rust caused by the pathogen *Puccinia striiformis* f. sp. *tritici*, and it is negatively regulated by tae-miR164 [63]. Recently, Fang et al. (2014) also reported the identification of *Oryza miR164-targeted NAC* (*OMTN*) genes that are targeted by the rice miR164 and their involvement in drought tolerance in rice [64]. In sunflower (*Helianthus annuus*), a recently evolved miR396 was found to target *HaWRKY6* in regulating early responses to temperature stress [65]. In a commonly cultivated apple cultivar (*Malus* x *domestica*), Md-miRNA156ab and Md-miRNA395 were identified to target *MdWRKYN1* and *MdWRKY26*, respectively, and mediate responses against the leaf spot fungus, *Alternaria alternate f. sp. mali* [66]. In rice, expression of miR5819 and miR5075 was induced by the rice blast fungus *Magnaporthe oryzae* and the miRNAs were found to target *OsbZIP38* and *OsbZIP27*, respectively [67]. In common bean (*Phaseolus vulgaris* L.), many identified *PvAP2-ERF* genes are also found to be the target of miRNAs from different plant species, with miR156, miR172 and miR838 likely to be involved in AP2/ERF regulation [68]. Therefore, in addition to the genetic regulation, epigenetic controls via histone and DNA modifications, as well as small RNA-mediated controls of the defense-related TFs, form an important molecular checkpoint in contributing to the overall accumulation of these transcriptional regulator families under stresses.

## 4. The Regulation of the Regulators at the Protein Level

The expression and production of defense-related proteins incurs metabolic cost and are closely connected to allocation of resources for plant growth and development [7,69,70,71]. Beyond transcriptional regulation, the regulation of defense-responsive transcription factors at protein level offers an added layer of control in achieving a balanced allocation of resources when plants are under stress and subjected to pathogen invasion. Nuclear translocation, protein-protein interaction and post-translational modification (PTM) of these TFs are required for their localization, activity and stability in mounting efficient stress-induced signaling cascades, as well as restoring basal gene expression when the stress condition subsides (Figure 2, steps 4–6).

### 4.1. Protein-Protein Interactions and Translocation

Nuclear localization is critical for TFs to activate or repress their target genes under stress conditions. In *Arabidopsis* and rice, many of the defense-related TFs were membrane-bound and released into the nucleus upon stresses [72] (Figure 2, step 4). For example, upon infection by *Ralstonia solanacearum* (a pathogenic bacterium), NtWRKY50 is induced to translocate to the nucleus in tobacco, leading to altered SA- and JA-mediated signaling pathways [73]. In rice, WRKY11 functions as a positive regulator for biotic and abiotic stresses. Subcellular localization assays showed that OsWRKY11 is translocated into the nucleus upon *Xanthomonas oryzae pv. oryzae* (*Xoo*) infection and drought stress [74]. In *Arabidopsis*, WRKY40 is a negative regulator of ABA signaling and in the presence of ABA, WRKY40 interacts with magnesium-protoporphyrin IX chelatase H subunit (CHLH)/ ABA receptor (ABAR) that is present in the outer chloroplast membrane [75]. Such cytosolic interaction led to the removal of WRKY40-mediated inhibition of gene expression in the nucleus. In response to different environmental stresses, two *Arabidopsis* bZIP factors, bZIP17 and bZIP28, translocated from the endoplasmic reticulum (ER) membrane to the Golgi apparatus where they were further processed for nuclear localization and activation of their downstream targets [76,77]. In contrast to bZIP17 and bZIP28, although bZIP60 is tethered to the ER (endoplasmic reticulum) membrane under normal conditions, ER-mediated cytoplasmic splicing of bZIP60 mRNA leads to the production of a bZIP60 variant that translocates directly into the nucleus for stress-responsive gene activation [78]. Similarly, stress-induced alternative splicing of the rice *OsDREB2B* was found to be an important regulatory mechanism for the nuclear localization of the functional variant of OsDREB2B [79]. A nuclear localization signal can also be found at the N-terminal NAC domain of many NAC TFs [28]. In *Medicago falcata*, an S-palmitoylated NAC TF was found to be dissociated from the membrane and translocated into the nucleus upon drought stress, thereby activating its downstream target for improved drought tolerance [80]. Another NAC TF, NTL6, is tethered to the plasma membrane under normal conditions. Upon cold stress, NTL6 (NTM (NAC with transmembrane motif1)-like 6) is induced and processed to relocate to the nucleus, activating *pathogenesis-related* (*PR*) gene expression [81]. Another two *Arabidopsis* NAC TFs, ANAC013 and ANAC017, were found to translocate from the ER membrane to the nucleus under oxidative stress [39,40]. Therefore, protease-mediated cleavage of membrane-bound transcription factors contribute an important regulatory mechanism for their spatial control and activation in response to stress. However, it is noted that for MYB and bHLH families of TFs, there are limited studies showing the release of membrane-bound TFs for DNA binding as a regulatory mechanism for the transcriptional activation of their targets.

In addition to protein localization, protein-protein interactions of the TFs in defense could mediate their DNA binding affinities and activities in targeting the activation/repression of their downstream defense-related genes through translocation of the TFs (Figure 2, step 5). In the cytoplasm, MYB30 interacts with the phospholipase A2s (AtsPLA2α (*Arabidopsis thaliana* secreted phospholipase A2-alpha)), leading to its translocation into the nucleus. Such protein-protein interaction with AtsPLA2α also rendered MYB30 incapable of activating hypersensitive response (HR) [82]. In response to biotrophic pathogen, nuclear translocation of the NPR1 (nonexpressor of pathogenesis-related genes1) protein enables its interaction with the TGACGTCA cis-element-binding protein (TGA) bZIP factors, leading to the activation of downstream SA-responsive genes [83,84]. In the presence of reactive oxygen species (ROS) upon pathogen attack, cytoplasmic AtbZIP10 (*Arabidopsis thaliana* basic leucine zipper 10) dissociated from lesions simulating disease resistance 1 (LSD1) in a complex and translocated into the nucleus for activation of defense responsive genes in HR [85]. Similarly, the activity of another bZIP factor in tobacco, BZI-1, in auxin signaling and pathogen response is mediated by its interaction with the cytosolic ankyrin-repeat protein (ANK1). Interaction between ANK1 and BZI-1 decreases BZI-1 translocation into the nucleus for target DNA binding and activation [86]. In JA signaling, the jasmonate ZIM (zinc-finger inflorescence meristem) domain (JAZ) proteins interact with MYC, preventing MYC from activating its downstream JA-responsive target genes [87,88]. Upon pathogen attack and insect herbivory, the production of JA-Ileu (isoleucine conjugate of jasmonic acid) leads to its interaction with JAZ and subsequent proteasomal degradation JAZ [89], thereby releasing MYC2 for target DNA binding and activation of JA-responsive genes. Homo- and hetero-dimer interaction among the closely related WRKY18, 40, and 60 were found to alter their DNA binding activities [90]. In *Arabidopsis*, a calmodulin binding NAC (CBNAC) TF is a transcriptional repressor of basal defense. The DNA binding activity of CBNAC was enhanced through its interaction with SNI1 (suppressor of npr1-1, inducible 1), thereby repressing the basal expression of *PR1* expression [91]. Therefore, in addition to spatial distribution of the TFs, specific protein-protein interaction forms an important regulatory node in mediating the activity as well as the translocation of transcription factors in plant defense, thereby maintaining the cost balance between plant growth and defense.

### 4.2. Post-Translational Modifications (PTMs)

Apart from spatial expression and localization, phosphorylation is one of the most-studied PTMs among the defense TFs leading to their activation or translocation in response to stress (Figure 2, step 5). In fact, protein kinase activity forms a key component in connecting various types of stress-sensing within a cell and the corresponding downstream signaling responses [92]. Some NAC TFs require phosphorylation to translocate into nucleus. For example, OsNAC4 is translocated into the nucleus in a phosphorylation-dependent manner and positively regulates hypersensitive cell death in response to pathogen in rice [93]. Within the WRKY TFs family, many WRKYs are activated by MAP kinases (MPKs) in respond to pathogen attack [94]. In *A. thaliana*, WRKY33 is phosphorylated via the MPK3/MPK6 signaling cascade, leading to camalexin induction upon *Botrytis cinerea* infection [34]. In defense against *Pseudomonas syringae*, AtWRKY25 and AtWRKY33 interact with MKS1 (MAPK substrate1) which in turns interacts with MPK4. Phosphorylation of MKS1 by MPK4 leads to the dissociation of the WRKYs in the complex, releasing them for activating their downstream targets for defense responses [33,95]. Similarly, in response to flagellin-derived flg22 peptide, AtERF104 is phosphorylated and dissociated from MPK6 [96]. In tobacco, NtWRKY1 is phosphorylated by a salicylic acid-induced protein kinase (SIKP), thereby increasing its affinity toward the W-box and ability to trigger HR cell death [97]. In response to abiotic stress, many bZIP TFs function through ABA-dependent pathways to which ABA activate the MAP kinases cascade, leading to phosphorylation of bZIP factors and expression of their downstream targets [98].

In plants, ubiquitin proteasome system (UPS) is responsible of the turnover of nearly 6% of the proteome, including many TFs [99,100]. OsWRKY6 and OsWRKY11 positively regulate defense and stress responses and their protein levels are controlled by UPS [74,101]. Recently, Matsushita et al. (2013) have shown that the C-terminal region of OsWRKY45 is critical not only for its transcriptional activity, but also UPS-dependent degradation [102]. Senescence is a form of programmed cell death that associates with the pathogen defense responses [103]. In *Arabidopsis*, WRKY53 is capable of activating the expression of specific senescence-related genes and other transcriptional regulators in defense [104]. AtWRKY53 was also found to interact with the HECT (homologous to the E6AP (E6-associated protein) carboxyl terminus) domain of E3 ubiquitin ligase protein (UPL5) and be ubiquitinated in vitro. Ectopic induction of *UPL5* expression in transgenics led to degradation of WRKY53, suggesting activity of WRKY53 is mediated by UPL5 through ubiquitin-mediated protein degradation [105]. In addition to the WRKY TFs, AtMYB30 is one of the major MYB factors in mediating defense against plant diseases [106]. AtMYB30 is a positive regulator of HR, and is responsible for increased resistance against bacterial pathogens [107]. In the nucleus, AtMYB30 was found to interact with the *Arabidopsis* RING (really interesting new gene)-type E3-ubiquitin-ligase MIEL1 (AtMYB30-Interacting E3 ligase1), directing AtMYB30 for proteasomal degradation [108]. Within the AP2/ERF family, activity of AtERF53 was also found to be mediated by ubiquitination through another RING domain ubiquitin E3 ligase, RGLG2 (RING domain ligase 2). Under normal condition, RGLG2 is translocated into the nucleus where it interacts with AtERF53 for proteolytic degradation. Under drought stress, AtERF53 is induced, activating its downstream targets expression [109]. NAC family TFs are also post-transcriptionally regulated by modification with N-acetylglucosamine or proteolytic processes [110]. In tomato, degradation of NAC1 is mediated through its interaction with the tomato ubiquitin ligase, SINA3 (seven in absentia3) [111]. Therefore, beyond protein phosphorylation, it appears that ubiquitination also forms a significant regulatory node in maintaining the homeostatic level of these defense TFs (Figure 2, step 6) and, hence, the cost balance in the production of their downstream targets.

## 5. The Potential of TFs in Agricultural Crop Improvement

Climate change, including changes in temperature, photoperiod, atmospheric CO_2_ concentration, precipitation, and wind patterns, can directly affect plant development or indirectly affect plant pathogens and insect pests [112]. Collected data from across the globe show that the Earth’s average temperature has increased about 1.11 degree Celsius during the 20th century. It is projected that this increase will result in the immigration of pathogens to new geographic regions and contacting with new plant hosts [113]. Moreover, global warming affects the survival of pathogen species in temperate and tropical regions. With increase in temperature, pathogen species in temperate areas can enhance their fitness and ability to cause diseases epidemics [114] because their temperature growth range is wider and more flexible than the temperature growth range of pathogen species in tropical regions [112]. Moreover, global warming causes longer growing seasons and provides more time available for pathogen reproduction [115]. It is estimated that there will be a reduction in the global yields for major crops, including wheat, rice, maize and soybean, with a range of 3–7% reduction for each degree Celsius increase in the global mean temperature [116]. Therefore, climate change poses a serious threat to crop production and global food security.

In the post-genomic era, the advancement of next generation sequencing (NGS) technologies has facilitated genome-scale genetic and functional studies of many agricultural crop species, especially in development and stress responses [117,118]. For instance, the availability of the rice genome sequence has facilitated the discovery the complex web of factors associated with salt tolerance mechanism in rice, paving ways for molecular breeding and genetic improvement of rice productivity against negative factors, such as drought and high soil salinity [119]. The potential threat of climate change to crop productivity is further compounded by the fact that development of cultivated crop species under optimized field conditions has led to the loss of valuable alleles that confer stress tolerance in their wild relatives [120,121,122]. For example, differential expression and alternative splicing of an emmer DRE-binding gene, *TdicDRF1* (*Triticum dicoccoides drought responsive element-binding factor 1*), was found to correlate to different drought-tolerant phenotypes in wild emmer wheat (*Triticum turgidum* ssp. *dicoccoides*) [123]. Sequence comparison of TdicDRF1 against its orthologs in hexaploid bread wheat, *Triticum aestivum* (TaDREB3A), and tetraploid durum wheat, *Triticum durum* (TdDRF1), revealed amino acid substitutions at the AP2 (APETALA2) domain, which in turn, altered its DNA binding specificity in vitro. Soybean cyst nematode (SCN; *Heterodera glycines* Ichinohe) is one of the most devastating soybean pests that causes considerable yield loss worldwide [124]. Genome wide association study (GWAS) in 235 accessions of the wild soybean (*Glycine soja*) has identified 43 accessions with resistance against SCN and candidate genes involved in SCN resistance [125], suggesting a rich wild genetic resource for stress-tolerant alleles that might have been lost or not selected during domestication of *Glycine max*. In defense against rice blast disease, caused by *Magnaporthe oryzae* (a fungal pathogen), wild rice (*Oryza rufipogon*) showed a genotype-specific mechanism against fungal infection when compared to cultivated rice (*Oryza sativa*). Expression of JA, ET biosynthetic genes, as well as some WRKY TFs, were more up-regulated in the wild rice than that in the cultivated rice upon *M. oryzae* infection [126]. Therefore, the availability of crop wild relatives (CWR) provides invaluable genetic resources for improvement of agricultural crops for biotic and abiotic stress tolerance in the fight against climate change [127,128]. With the advance of genomic technologies, growing wild germplasm collections, and vast amounts of sequence data for cultivated crops and their CWR [128,129,130,131,132,133], now would be the time to re-examine and explore wild relatives of crop plants for the lost or alternative alleles that may contribute to better stress tolerance observed in most wild crop species.

## 6. Conclusions and Perspectives

**Figure 3:**
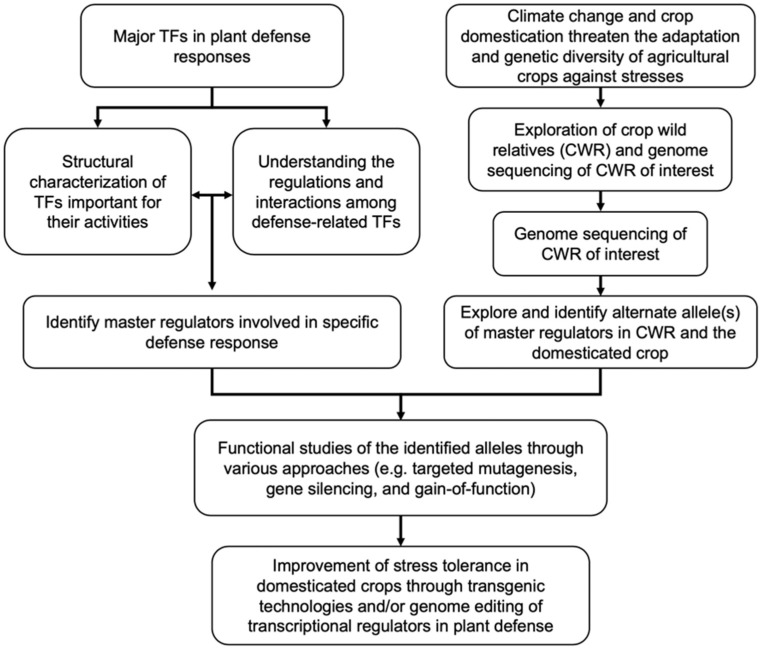
Flow diagram illustrating a strategy for genetic improvement of domesticated crops through genetic engineering of transcriptional regulators in plant defense. Understanding the structural characteristics of TFs that are important in determining their expression at transcriptional (auto-/cross-regulations), post-transcriptional (miRNA, alternative splicing) and post-translational (protein-protein interactions, localization, modification, and stability) will allow the identification of candidate master regulators for genetic improvement (left). Climate change and domestication of crops will have negative impacts on food security due to limited adaptation and genetic diversity of agricultural crops against stresses. In parallel, with the advance of genome technologies and identification of crop wild relatives (CWR), genome sequencing of CWR will permit the exploration of alternative alleles of potential master regulators in the wild relatives (right). Integrating such information will facilitate the selection of transcriptional regulators for functional studies using various approaches and subsequent improvement of stress tolerance in domesticated crops through transgenic technologies and/or marker assisted breeding.

In this review, we discussed various levels of molecular mechanisms underlying the possible regulatory nodes controlling the expression and activity of representative transcriptional regulator families in plant defense (Figure 3). Although many studies have been performed to identify various genes involved in stress resistance in CWR and introduce them into cultivated crops, research focusing on TF-based modifications is still scarce. With climate change quickly becoming a major threat to food production in the 21st century, there is a tremendous potential of using CWR to discover alternative alleles of these TF families. A recent review by Rabara et al. (2014) has provided a comprehensive view of such potential in manipulating TFs for crop genetic improvement [134]. Finally, the advancement of sequencing technologies in recent years has provided researchers with whole genome information of many agricultural crops and their wild relatives, thereby facilitating the identification of beneficial wild alleles in CWR. Coupled with genome editing technologies for targeted gene modification and transgenic technologies, we envision that modulating the regulation and activities of transcriptional regulators could play a pivotal role toward the genetic improvement of domesticated crop cultivars, especially in stress tolerance and defense responses.

## Figures and Tables

**Table 1:** The major transcription factor families involved in plant defense and their numbers.

Transcription Factor Family	Number of Genes Identified in the Plant Genomes ^1^				
	*Arabidopsis thaliana*	*Tritcum asetivum*	*Oryza sativa*	*Zea may*	*Glycine max*
AP2/ERF ^2^	146	230	158	213	365
AP2 subfamily	18	43	16	29	59
ERF + DREB subfamily	122	179	138	182	301
RAV subfamily	6	8	4	2	5
MYB	205	482	191	283	500
bHLH	153	321	156	202	355
NAC	113	260	141	133	180
WRKY	72	170	102	123	185
bZIP	74	185	96	129	152

AP2/ERF (APETALA2/ethylene responsive factor), bHLH (basic helix-loop-helix), MYB (myeloblastosis related), NAC (no apical meristem (NAM), Arabidopsis transcription activation factor (ATAF1/2), and cup-shaped cotyledon (CUC2)), WRKY, and bZIP (basic leucine zipper). ^1^ The number of genes is obtained by counting the number of gene IDs listed in the plant transcription factor database v4.0, excluding gene IDs of alternative transcripts [16]; ^2^ For the AP2/ERF family, the number of genes from three subfamilies were also listed, including AP2, ERF + DREB (dehydration-responsive element-binding protein), and RAV (related to abscisic acid insensitive3/viviparous1) subfamilies.
